# Expression Patterns and Subcellular Localization of Carbonic Anhydrases Are Developmentally Regulated during Tooth Formation

**DOI:** 10.1371/journal.pone.0096007

**Published:** 2014-05-01

**Authors:** Claes-Göran Reibring, Maha El Shahawy, Kristina Hallberg, Marie Kannius-Janson, Jeanette Nilsson, Seppo Parkkila, William S. Sly, Abdul Waheed, Anders Linde, Amel Gritli-Linde

**Affiliations:** 1 Department of Oral Biochemistry, Sahlgrenska Academy at the University of Gothenburg, Göteborg, Sweden; 2 Department of Oral Biology, Minia University, Minia, Egypt; 3 Department of Chemistry and Molecular Biology, University of Gothenburg, Göteborg, Sweden; 4 School of Medicine and BioMediTech, University of Tampere and Fimlab, Tampere University Hospital, Tampere, Finland; 5 Saint Louis University School of Medicine, Saint Louis, Missouri, United States of America; University of Palermo, Italy

## Abstract

Carbonic anhydrases (CAs) play fundamental roles in several physiological events, and emerging evidence points at their involvement in an array of disorders, including cancer. The expression of CAs in the different cells of teeth is unknown, let alone their expression patterns during odontogenesis. As a first step towards understanding the role of CAs during odontogenesis, we used immunohistochemistry, histochemistry and in situ hybridization to reveal hitherto unknown dynamic distribution patterns of eight CAs in mice. The most salient findings include expression of CAII/*Car2* not only in maturation-stage ameloblasts (MA) but also in the papillary layer, dental papilla mesenchyme, odontoblasts and the epithelial rests of Malassez. We uncovered that the latter form lace-like networks around incisors; hitherto these have been known to occur only in molars. All CAs studied were produced by MA, however CAIV, CAIX and CARPXI proteins were distinctly enriched in the ruffled membrane of the ruffled MA but exhibited a homogeneous distribution in smooth-ended MA. While CAIV, CAVI/*Car6*, CAIX, CARPXI and CAXIV were produced by all odontoblasts, CAIII distribution displayed a striking asymmetry, in that it was virtually confined to odontoblasts in the root of molars and root analog of incisors. Remarkably, from initiation until near completion of odontogenesis and in several other tissues, CAXIII localized mainly in intracellular punctae/vesicles that we show to overlap with LAMP-1- and LAMP-2-positive vesicles, suggesting that CAXIII localizes within lysosomes. We showed that expression of CAs in developing teeth is not confined to cells involved in biomineralization, pointing at their participation in other biological events. Finally, we uncovered novel sites of CA expression, including the developing brain and eye, the olfactory epithelium, melanoblasts, tongue, notochord, nucleus pulposus and sebaceous glands. Our study provides important information for future single or multiple gene targeting strategies aiming at deciphering the function of CAs during odontogenesis.

## Introduction

A simple, yet crucial reaction, the reversible hydration of carbon dioxide into bicarbonate and protons (CO_2_+ H_2_O ⇔ HCO_3_^−^+H^+^), takes place in virtually all living forms and is catalysed by carbonic anhydrases (CAs). In mammalian cells, CAs are thus involved in several biological processes that directly or indirectly use components of this reaction, including respiration, pH regulation, secretion of electrolytes, bone resorption and biomineralization as well as HCO_3_^–^ dependent metabolic processes [1]–[3]. Because of their involvement in a range of disorders, CAs are important therapeutic targets [1]–[3]. In vertebrates CAs are encoded by the *α-CA* gene family, and mammalian genes code for 16 different isoforms among which 13 are catalytically active zinc metalloenzymes. These include CA I, CA II, CA III, CA IV, CA VA, CA VB, CA VI, CA VII, CA IX, CA XII, CA XIII, CA XIV and CA XV (CA XV is not expressed in humans). The remaining three CAs that are devoid of catalytic activity are known as CA-related proteins (CARPs) and include CARP VIII, CARP X and CARP XI [4], [5]. The mammalian CAs display differences in their catalytic activities, sensitivity to inhibitors as well as tissue and subcellular localization. CA I, CA II, CA III, CA VII and CA XIII are found in the cytosol, membrane-bound isoenzymes include CA IV, CA IX, CA XII, CA XIV and CA XV, whereas CA VA and CA VB localize in mitochondria. CA VI is a secreted isoform expressed in the alimentary tract and in several glands [2], [3], [5], [6]. However, *Car6^−/−^* mice lacking the function of CA VI have no major cytological alterations in these organs, suggesting compensation by other CAs in those tissues [7].

CA II is the most widely distributed enzyme [3], [8]–[12] and the importance of its function is reflected in human subjects with mutations in the *CA2* gene leading to loss of function. The affected individuals develop osteopetrosis, brain calcifications and renal tubular acidosis, a condition known as CA II deficiency syndrome (CADS) [3]. *Car2* mutant mice fail to fully phenocopy the defects in humans with CADS, as they only show stunted growth, renal tubular acidosis and mild bone defects [13], [14]. CA III/*Car3* (protein/mRNA) are expressed in several developing and adult tissues, including mesodermal precursors and their derivatives, the gastrointestinal tract and brain [3], [5], [10], [15]. However, CA III deficiency in mice results only in a mild muscle defect [16], pointing at some compensatory events by other isoenzymes. CA XIII shows a widespread distribution in adult human and murine tissues [17].

The mild phenotypes observed in mice deficient in the cytosolic CAs are also encountered with the membrane-bound ones. Nevertheless, previous investigations revealed some tissue-specific functions for these isoenzymes. Insights from mice deficient in CA IV or lacking both CA IV and CA XIV functions suggest that both enzymes are involved in extracellular buffering in the central nervous system and that CA XIV is required for retinal function [3], [18]. Importantly, human mutations in *CA4* generate *retinitis pigmentosa* characterized by degeneration of photoreceptors [3], [19]. CA IV has also been shown to act as the principal CO_2_ taste sensor [20] and to be invloved in the human and mouse reproductive systems [21].

Besides the stomach where it is present at high levels, CA IX is also produced in the brain and gut [22]. Mice deficient in CA IX function display gastric epithelial hyperplasia [23] and brain vacuolar degenerative changes leading to functional anomalies [24]. In addition to the central nervous system CARP XI is expressed in other organs, and CARP XI protein and mRNA have been shown to be upregulated in several human cancers [4].

The overlapping expression patterns of several CAs in a wide range of tissues and organs constitute a hurdle for studies aiming at deciphering the specific roles of a given isoform in a specific tissue. In addition, the role of CAs during embryonic and fetal development is still largely unexplored. There is also a need for a good experimental system, different from cell cultures, to study the role of CAs and test potential CA activators and inhibitors. One such a system is the developing tooth which enables studies of the biological mechanisms regulating morphogenesis, cellular differentiation and biomineralization [25]–[29]. Furthermore, mouse developing teeth are also amenable to culture in an organ culture system in vitro, thus providing conditions that best mimick the in vivo environment.

The developing tooth is made of an epithelium and a mesenchyme which interact to drive its development from initiation until completion. At the bell stage of odontogenesis (tooth formation) the different cellular components of a tooth become distinguishable. At this stage, the developing tooth consists of an epithelial enamel organ and a dental mesenchyme. The epithelial enamel organ includes a proliferating inner dental epithelium that gives rise to proliferating preameloblasts which differentiate into secretory ameloblasts, cells that produce the enamel matrix. After completion of enamel secretion, these cells undergo a transition stage followed by differentiation into maturation-stage ameloblasts (MA), the function of which is crucial for the formation of the mature enamel. The other components of the epithelial enamel organ are cells of the stratum intermedium, the stellate reticulum and the outer dental epithelium. At the maturation stage, these three cell types form the papillary layer abutting MA, whereas the remaining stellate reticulum and outer dental epithelium surround the developing tooth’s crown. The dental mesenchyme consists of the dental papilla mesenchyme (DP) and the dental sac. A subset of cells of the DP gives rise to proliferating preodontoblasts which ultimately differentiate into odontoblasts producing predentin and dentin components, whereas postnatally the remaining cells of the DP form the dental pulp. Derivatives of the dental sac mesenchyme include cementoblasts, alveolar bone osteoblasts as well as fibroblasts in the periodontal ligament.

Previous histochemical studies in rodent teeth showed robust CA activity mainly in MA, the PL, preodontoblasts and the stratum intermedium [30], [31]. Later on, immunohistochemistry, RT-PCR as well as northern blot analyses revealed high levels of expression of CA II/*Car2* and CA VI/*Car6* during the maturation stage of enamel formation (amelogenesis) in MA and in cellular extracts from the entire epithelial enamel organ [26], [32]–[34], and that CA II protein is also present in transition-stage ameloblasts [33]. More recently, with the exception of *Car5a*, RT-PCR showed that mRNAs of all mouse CA isoforms are expressed in extracts derived from the epithelial enamel organ during the secretory stage of amelogenesis, with *Car2*, *Car6*, *Car9*, *Car11*, *Car12* and *Car13* displaying the highest levels of expression [35].

It remains however unclear as to which cells of the epithelial enamel organ express those isoforms. In addition, the distribution of CAs in the epithelial enamel organ as well as in other tooth components during the different stages of tooth development is still unknown. Furthermore, as regards secretory ameloblasts, the issue is still unresolved, since immunological [33] and histochemical [31] studies were unable to detect CA II protein or CA activity in these cells, whereas *Car2* mRNA was detected in epithelial enamel organ extracts at the secretory stage of amelogenesis [34], [35].

As a first step towards understanding the role of the different CA isoforms during odontogenesis, we thus performed a systematic study of the expression patterns of CAII/*Car2*, CA III, CA IV, CA VI/*Car6*, CA IX, CARP XI, CA XIII and CA XIV during mouse tooth development. In addition, novel sites of CA expression in non-dental tissues are also briefly described, especially during embryogenesis and shortly after birth, as most previous studies focused on adult organs. We show that CAs display dynamic patterns of expression during tooth development. Importantly, we uncovered hitherto unknown subcellular localization of several CAs.

## Materials and Methods

### Ethics Statement

The experiments were reviewed and approved by the Animal Research Ethics Committee in Göteborg, Sweden (Dnr. 230-2010, 174-2013).

### Specimen Preparation

Embryonic day (E) 12.5 mouse embryos as well as mouse heads at E13.5, E14.5, E18.5 and 1 day postpartum (dpp) were fixed overnight at 4°C in 4% paraformaldehyde (PFA) and processed for paraffin embedding. Twelve dpp mice were fixed with 4% PFA by perfusion through the left heart ventricle, after which the heads were post-fixed overnight in PFA. After demineralization in PBS containing 2.5% PFA and 12% EDTA, pH 7.4 for 4–6 weeks at 4°C, the specimens were processed for paraffin embedding. Because the above demineralization mixture was not always conducive to immunostaining with the goat anti-CA XIII antibody (especially after a long demineralization period), other specimens (12 dpp) were fixed overnight in 95% ethanol containing 1% glacial acetic acid and demineralized for 5 days in a mixture containing 4% PFA, 10% glacial acetic acid and 0.85% NaCl. Kidneys, submandibular glands, stomachs and cerebella from adult mice were fixed in 4% PFA and processed for paraffin embedding.

### Immunohistochemistry and *in situ* Hybridization

The primary antibodies used and their dilutions are listed in Table 1. Briefly, after dewaxing and antigen unmasking in 10 mM citrate buffer (for the goat anti-CA IX, 1 mM EDTA, pH 8 was used instead of citrate buffer), the sections were treated for 10 min with 3% H_2_O_2_ in methanol. After blocking of non-specific binding sites [5% normal serum in PBS containing 0.1% bovine serum albumin and 0.1% Triton-X-100 (PBS/BSA/Triton-X-100)], the primary antibodies, diluted in PBS/BSA/TritonX-100 were applied overnight at 4°C. For immunostaining using primary antibodies produced in goat, BSA and other bovine products were avoided as bovine immunoglobulins that may contaminate them react with anti-goat secondary antibodies. Thus, the blocking step was done using normal rabbit serum (NRS; 5%) in PBS/Triton-X-100, and primary antibodies were diluted in 1% NRS in PBS/Triton-X-100. The following steps, including incubations with biotinylated secondary antibodies and vizualization of antigenic sites, were done using the Vectastain ABC kit (Vector Laboratories) according to the manufacturer’s protocol. Immunostaining using biotinylated tyramide amplification system was as previously described [36]. For generation of the riboprobes, pBluescript vectors containing mouse *Car2* (the first 1542 bp) and *Car6* (the first 1372 bp) cDNA sequences ligated at the *Not1*/*Sal1 s*ites were linearized with *XhoI* and transcribed with T3 RNA polymerase. In situ hybridization with ^35^S-UTP-labelled riboprobes was done on sections from paraffin-embedded specimens as described previously [37].

**Table 1:** Antibodies used.

Antibody	Source	Catalogue no./reference	Dilution fornon-demineralizedtissues	Dilution for demineralizedtissues
Rabbit anti-CA II (H-70)	Santa Cruz Biotechnology	sc-25596	1/750	1/1,000
Rabbit anti-CA III; antigen affinity-purified	ProteinTech	15197-1-AP	1/1,000 (1/1,200 forE12.5–E14.5 embryos)	1/1,000
Goat anti-CA IV; antigen affinity-purified	R&D Systems	AF-2414	1/1,000	1/1,000
Rabbit anti-CA VI (1-18-R; antigen affinity-purified	Santa Cruz Biotechnology	sc-27893-R	1/2,000	1/3,000
Rabbit anti-CA IX (M-100)	Santa Cruz Biotechnology	sc-25600	1/250	1/250
Goat anti-CA IX; antigen affinity-purified	R&D Systems	AF-2344	1/3,000	1/3,000
Rabbit anti-CARP XI (H-50)	Santa Cruz Biotechnology	sc-67333	1/600	1/1,000
Goat anti-CA XIII (K-16); antigen affinity-purified	Santa Cruz Biotechnology	sc-sc-54768	1/500 without tyramide or1/2,000 withtyramide amplification	1/500 or 1/1,500 (with andwithout tyramide)
Rabbit anti-mouse CA XIII antiserum	S. Parkkila	Lehtonenet al. [17]	1/4,000	1/4000
Rabbit anti-mouse CA-XIV antiserum	A. Wahed/W.S. Sly	Ochrietoret al. [43]	1/8,000	1/8,000
Goat anti-CA XIV (N-19); antigen affinity-purified	Santa Cruz Biotechnology	sc-17256	1/200	1/200
Rat monoclonal anti-LAMP-2(clone 6A430)	Santa Cruz Biotechnology	sc-71492	1/1,000	1/1,000 with tyramideamplification
Rat monoclonal anti-LAMP-1(Clone 1D4B)	Santa Cruz Biotechnology	sc-19992	1/40,000	1/40,000 (high conc.)1/50,000 (low conc.)

### Single, Double and Triple Immunofluorescence

Preparation of sections up to incubation in the presence of the first primary antibody were made as described above. The sections were incubated overnight with the goat anti-CA XIII antibody (diluted 1∶2000 in PBS containing 1% NRS and 0.1% Triton-X-100). After washing and incubation for 1 hour at room temperature (RT) with horseradish peroxidase (HRP)-conjugated rabbit anti-goat IgG (Thermo Scientific), the sections were treated with the Alexa Fluor 488 tyramide conjugate (InVitrogen) according to the manufacturer’s instructions. Slides with sections stained solely with the CA XIII antibody were mounted after washing the tyramide conjugate. Slides with sections that were intended for double and triple immunostaining with CA XIII and LAMP-1 and LAMP-2 antibodies were washed in PBS after the tyramide reaction. After blocking of peroxidase and non-specific binding sites, the sections were incubated overnight at 4°C with the LAMP-1, LAMP-2 or combined LAMP-1 and LAMP-2 antibodies, both diluted 1∶1000 in PBS/BSA/Triton-X-100. Subsequent to washing and incubation with a biotinylated secondary antibody (rabbit anti-rat), the sections were washed and incubated with Streptavidin Alexa Fluor 594 conjugate (InVitrogen) according to the manufacturer’s (InVitrogen) instructions. After a final wash in PBS, the slides were mounted with ProLong Gold anti-fade reagent (InVitrogen). For sections stained with LAMP-1 or LAMP-2 antibodies without CA XIII immunostaining, after dewaxing, blocking of endogenous peroxidase and non-specific binding sites, the sections were incubated overnight at 4°C in either LAMP-1 or LAMP-2 antibodies diluted as described above. The sections were thereafter incubated with the biotinylated rabbit anti-rat, washed and incubated with the streptavidin-AlexaFluor 594 conjugate and processed for mounting as described above. The negative controls were processed in parallel to the test slides but without primary antibodies. For CA III immunofluorescence, the sections were incubated overnight at 4°C with the CA III antibody (diluted at 1∶1500 in PBS/BSA/Triton-X100). Following washes, the sections were incubated with HRP-conjugated goat anti-rabbit, treated with the Alexa Fluor 488 tyramide conjugate and processed as described above.

### Histochemistry for Carbonic Anhydrase and β-galactosidase Activities

Generation of *K14-Cre; R26R* reporter mice and β-galactosidase histochemistry were as described previously [38]. However for β-galactosidase staining, the jaws were fixed in 2% PFA in PBS, decalcified in 10% EDTA, pH 7.3 for 5–6 weeks after which, they were cryoprotected overnight at 4°C in PBS containing 30% sucrose, embedded in OCT compound and sectioned (12 µm-thick sections) in a cryostat prior to histochemistry. For carbonic anhydrase histochemistry, jaws from 12 dpp mice were processed as previously described [31].

## Results

Although the specificity of the antibodies againsts the different CA isoforms has been established by the manufacturers or previous studies, we performed additional testing by using tissues known to produce CAs as positive controls (Fig. S1). These include the choroid plexus for CA II [39], the notochord and the nucleus pulposus for CAIII [15], proximal kidney tubuli for CA IV [40] and CA XIV [41], serous acini of the submandibular salivary gland for CA VI [7], the gastric mucosa for CA IX [22], cerebellar Purkinje neurons for CARP XI [42] and kidney ducts and tubuli for CA XIII [17]. The specificities of the CA IV antibody as well as the rabbit antisera against mouse CA XIII and mouse CA XIV have also been validated previously [17], [21], [43]. It is noteworthy that several antibodies generated in the same species but targeting different CA isoforms (as will be shown) or other proteins (data not shown) gave different expression patterns in dental and non-dental tissues, thus providing an additional control. No immunostaining was detected in sections used as negative controls where the primary antibodies were omitted, with the exception of background staining of the enamel or dentin matrices, especially in demineralized teeth. This is likely due to non-specific binding of reagents to these extracellular matrices which we found to be increased after antigen unmasking (Fig. S2F−J & data not shown). Similarly, sections in which the non-affinity-purified primary antisera were replaced by rabbit non-immune serum showed no staining in cells. However, biotinylated tyramide amplification used in sections from 1 dpp teeth as controls for sections stained with the goat-anti-CA XIII led to a homogeneous cytoplasmic background staining of ameloblasts and odontoblasts (Fig. S2K–M). In contrast to the antigen affinity-purified goat anti-CA XIII and goat anti-CA XIV, the rabbit antisera against CA XIII and CA XIV generated a yellowish/brownish background staining in the extracellular spaces of several tissues, including teeth. This may be due to some non-specific staining with these non-affinity-purifed antisera. Nevertheless, these antisera visualized patterns of specific cellular staining similar to those with the antigen-affinity-purified antibodies (data not shown for CA XIV). Both CA IX antibodies gave similar expression patterns (Fig. S1F, G & data not shown).

The data presented below concern to a large extent the developing molar teeth, however we have observed similar expression patterns of CA isoforms in developing incisor teeth as well. Finally, in addition to immunohistochemistry, we used in situ hybridization to show *Car2* and *Car6* mRNA expression patterns.

### Expression Patterns of CA Isoforms during the Different Stages of Odontogenesis

#### Tooth initiation

**Figure 1:**
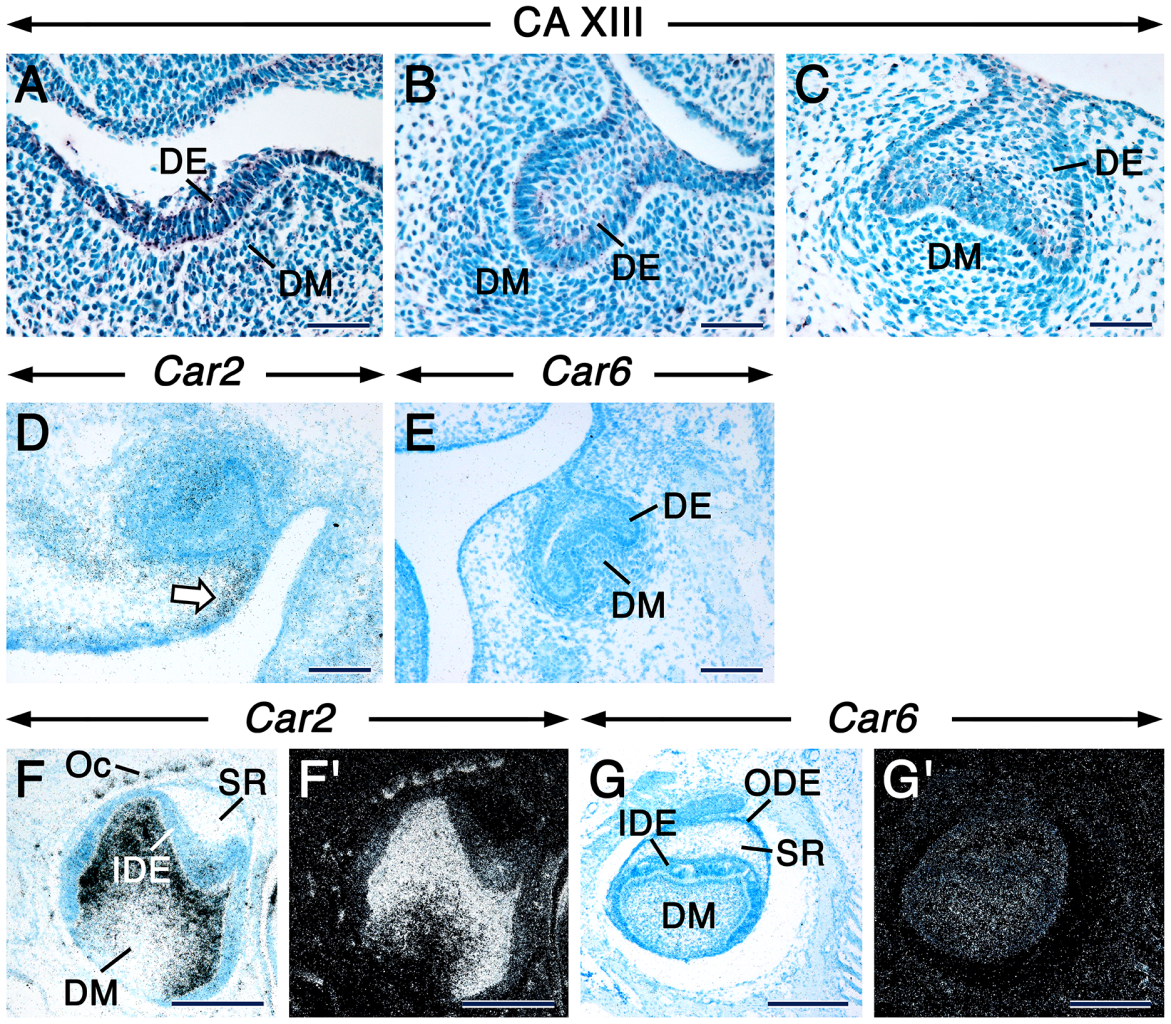
CA XIII, *Car2* and *Car6* distribution during early tooth development. Sections from E12.5 (A), E13.5 (B) and E14.5 (C–E) embryo heads at the levels of developing first molar at the placodal (A), bud (B) and cap (C–E) stages. Mandibular (A–C, E) and maxillary (D) molar sections. Sections of third molars at 12 dpp at the level of their less differentiated cusps (F–G’). Immunostaing showing CA XIII distribution (A–C). Dark magenta color indicates the sites of immunostaining. CA XIII appears as intracellular punctae. In situ hybridization showing expression of *Car2* (D, F, F’) and *Car6* (E, G, G’). Signals appear as shiny dots in dark-field images (F’, G’). Bright-field images (D, E, F, G). Robust signals appear as black silver grains in bright-field images. In addition to the dental mesenchyme, the mesenchyme at the periphery of the tooth expresses *Car2* (arrow in D). Abbreviations: DE, dental epithelium; DM, dental mesenchyme; IDE, inner dental epithelium; ODE, outer dental epithelium; OC, osteoclasts; SR, stellate reticulum. Scale bars: 50 µm (A–C), 100 µm (D, E), 200 µm (F, F’, G, G’).

The earliest histological evidence of tooth development is the appearance of an ectodermal placode, a local thickening of the oral epithelium. At embryonic day 12.5 (E12.5), only CA XIII was detectable in the molar tooth placodes and the subjacent dental mesenchyme (Table S1). Intriguingly, the CA XIII immunostaining was portrayed by strongly stained intracytoplasmic punctae/vesicles (Fig. 1A), wich were also evident in other non-dental embryonic and adult tissues (Fig. S1I & data not shown).

#### Tooth bud stage

At E13.5, the embryonic tooth consists of an epithelial bud surrounded by a condensed dental mesenychyme. At this stage, only CA II, CA VI and CA XIII proteins were detectable in the dental epithelial bud and associated dental mesenchyme (Table S1). However, in contrast to CA II and CA VI, CA XIII protein was visualized as distinct intracytoplasmic punctae in both the dental epithelium and mesenchyme (Fig. 1B).

#### Tooth cap stage

At E14.5, the tooth reaches the cap stage during which the dental epithelium forms a cap enclosing the dental papilla mesenchyme. Only CA II, CA VI and CA XIII (Fig. 1C, Table S1) proteins were detected at this stage in both the dental epithelium and mesenchyme. Remarkably, CA XIII protein continued to localize in punctae. At this stage, *Car2* mRNA was clearly detectable in the dental epithelium and mesenchyme, but the highest levels of expression were confined to a small domain of non-dental mesenchyme at the periphery of the tooth primordium (Fig. 1D). By contrast, a faint *Car6* signal was detectable in the dental epithelium and mesenchyme (Fig. 1E).

#### Tooth bell stage and early cytodifferentiation

At the bell stage of odontogenesis, developing molar teeth exhibit prominent principal cusps which are first to develop, whereas their minor cusps are either not yet formed or are at the early stages of their development. Within a given cusp, there is a gradient of cytodifferentiation of cells of the ameloblastic (inner dental epithelium, preameloblasts and secretory ameloblasts) and the odontoblastic (preodontoblasts, newly differentiated odontoblasts and odontoblasts, the latter being the most mature cells producing both predentin and dentin components) lineages, with the most differentiated cells localizing at its tip and the less differentiated ones situated along its slopes [25]. At E18.5 and 1 dpp, in addition to the dental papilla mesenchyme and the other components of the epithelial enamel organ (see Introduction), the principal cusps of the first molars contain newly differentiated odontoblasts that have secreted the first layer of predentin matrix as well as preameloblasts. At 12 dpp, the least developed cusps of the third molars are at the early bell stage and consist of an epithelial enamel organ and a dental papilla mesenchyme devoid of differentiated cells.

The incisor teeth of rodents continue to grow during the lifetime of the animal, owing to the presence of an active stem cell region [44], [45]. The tooth thus undergoes all stages of development along its anterior-posterior axis, with the most advanced stages located anteriorly and the less developed stages located posteriorly [46]–[48]. At specific levels posteriorly, the incisor of a 12 dpp mouse is at a developmental stage similar to that of molars at E18.5-1 dpp.

**Figure 2:**
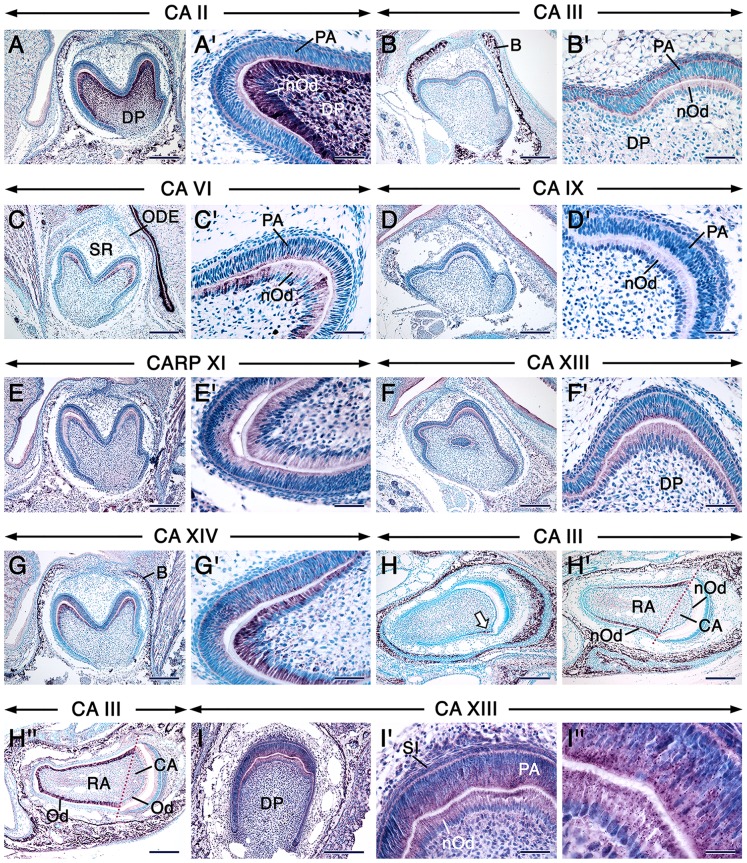
Carbonic anhydrase distribution during the bell/cytodifferentiation stage of odontogenesis. Sections of first molars at 1 dpp (A–G’) and posterior segments of incisors at 12 dpp (H–I”) after immunostaining for the different carbonic anhydrases as indicated on the panels. Dark magenta color indicates the sites of immunoreactivities. The dental papilla mesenchyme and newly differentiated odontoblasts (nOd) show robust CA II staining. CA III is asymmetrically expressed in incisors (H–H”). The arrow in H points at the first odontoblasts that differentiate in the root analog of the incisor which initiate robust expression of CA III. CA VI, CARP XI and CA XIV are expressed in preameloblasts and newly differentiated odontoblasts but not in the dental papilla mesenchyme. Intracytoplasmic punctae/vesicles show strong CA XIII immunostaining (F, F’, I–I”). A’, B’, C’, D’, E’, F’, G’ and I’ are high magnification views of A, B, C, D, E, F, G and I, respectively. I’’ is a high magnification view of I’. Abbreviations: B, alveolar bone; CA, crown analog part of the incisor; DP, dental papilla mesenchyme; Od, odontoblasts, ODE, outer dental epithelium; PA, preameloblasts; RA, root analog part of the incisor; SR, stellate reticulum; SI, stratum intermedium. Scale bars: 200 µm (A–I), 50 µm (A’–G’, I’), 20 µm (I”).

The distribution of CA proteins in tooth-forming cells at the cytodifferentiation stage of tooth formation is summarized in Table S1. In incisors and molars, the dental papilla mesenchyme and newly differentiated odontoblasts underwent an impressive boost in CA II/*Car2* protein and mRNA expression (Fig. 2AA’& data not shown). In fact, the boost in CA II/*Car2* expression levels in the dental papilla mesenchyme occurred during the early bell stage, before odontoblast differentiation (Fig. 1FF’ & data not shown). Weak staining for CA III was detectable in preameloblasts and newly differentiated odontoblasts of molars (Fig. 2BB’). Interestingly, a closer look at different anterior-posterior levels of incisors revealed that the distribution of CA III was asymmetric, in that it was virtually confined to odontoblasts and newly differentiated odontoblasts in the root analog (Fig. 2H–H”). This pattern continued to be evident at later stages of incisor development (Fig. S2B & data not shown). No CA IV immunoreactivity was detected in tooth primordia at this stage. After being weakly expressed in undifferentiated dental cells at the early bell stage (Fig. 1 GG’ & data not shown), CA VI/*Car6* became readily detectable in preameloblasts and newly differentiated odontoblasts at cytodifferentiation stage (Fig. 2CC’ & data not shown). These cells showed a faint CA IX staining (Fig. 2DD’) and patent CARP XI, CA XIII and CA XIV immunoreactivities. However, in contrast to CARP XI (Fig. 2EE’) and CA XIV (Fig. 2GG’), CA XIII protein was localized in strongly stained intracytoplasmic punctae together with a weaker staining in the rest of the cytoplasm (Fig. 2FF’& I–I”). The cytoplasmic CA XIII staining was likely non-specific, since it was detected in negative controls (Fig. S2K & L).

#### Advanced tooth development

At 12 dpp, the epithelial enamel organ of the crowns of the first and second molars has undergone extensive changes and root development is underway, whereas the most advanced cups in the third molars have started enamel secretion. In the first and second molars, ameloblasts in most cusps have ceased enamel secretion and transformed into maturation-stage ameloblasts after a brief transition stage during which secretory ameloblasts become reduced in height. Maturation-stage ameloblasts are known to modulate between two cell types, the ruffle-ended and the smooth-ended maturation-stage ameloblasts [49], [50]. At the transition and maturation stages, cells of the stratum intermedium, stellate reticulum and outer dental epithelium form the papillary layer abutting maturation-stage ameloblasts [51]. After complete demineralization, the entire mature enamel layer disappears leaving only the so-called enamel space.

**Figure 3:**
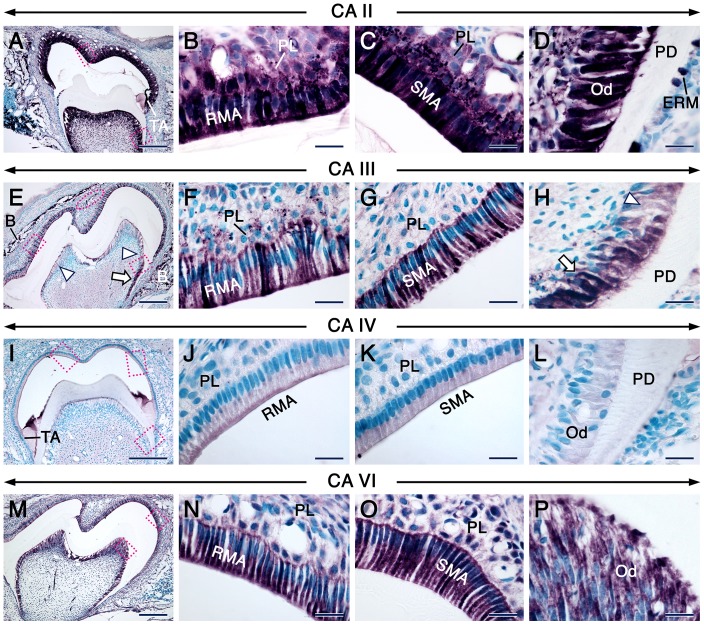
CA distribution patterns in postnatal molars at the maturation stage of enamel formation. Immunohistochemistry (IHC) showing the distribution patterns (Magenta color) of CA II, CA III, CA IV and CA VI as indicated on the panels. Ruffle-ended (RMA in B, F, N) and smooth-ended (SMA in C, G, O) maturation-stage ameloblasts show robust immunostaining representing CA II, CA III and CA VI. CA IV (J) is enriched in the ruffled border of the RMA whereas the SMA exhibit a homogeneous CA IV staining (K). CA II (D), CA IV (L) and CA VI (P) immunostaining of odontoblasts (Od). Odontoblasts in the root (arrows in E and H) display strong staining portraying CA III, whereas their crown counterparts display nearly background levels of immunostaining (arrowheads in E and H). The occlusal outer dental epithelium displays strong staining for CA III (red cercle in E). The papillary layer (PL) adjacent to both RMA and SMA display strong CA II immunostaining (B, C). The PL adjacent to the RMA (F) shows stronger staining for CA III than the PL adjacent to the SMA (G). Images in B, D F, H J, K, L, N and P are high magnification views of the areas indicated in A, E, I and M, respectively. Because some sections of molars at the maturation stage of enamel formation do not always comprise SMA, the images shown in C, G and O are taken either from the same molar, but a few sections away or from a section of another molar after IHC under the same conditions. Additional abbreviations: B, bone; ERM, epithelial rests of Malassez; PD, predentin/dentin; TA, transition-stage ameloblasts. Scale bars: 200 µm (A, E, I, M), 20 µm (B–D, F–H, J–L, N–P).

**Figure 4:**
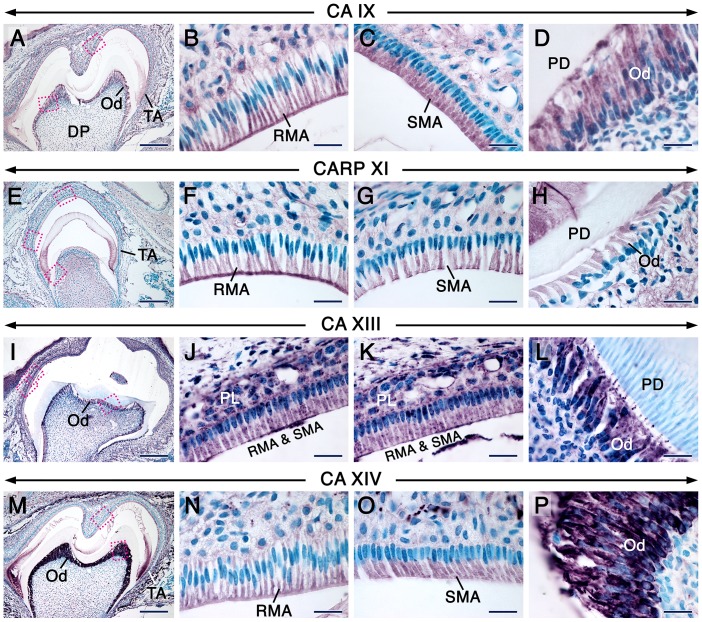
CA immunohistochemistry in postnatal molars at the maturation stage of enamel formation. Immunohistochemistry showing the distribution patterns of CARP XI, CA IX, CA XIII and CA XIV in sections of molars at 12 dpp as indicated on the panels. The latter three isoforms were detected with the Goat anti-CA IX, rabbit anti-mouse CA XIII antiserum and goat anti-CA XIV. CA IX (B) and CARP XI (F) are enriched in the ruffled border of ruffle-ended maturation-stage ameloblasts (RMA). Smooth-ended maturation-stage ameloblasts (SMA) exhibit a homogeneous staining portraying CA IX (C) and CARP XI (G). CA XIV immunostaining is homogeneous in RMA (N) and SMA (O). Odontoblasts (Od) express CA IX (D), CARP XI (H) and CA XIV (P). CA XIII immunostaining is strong in intracytoplasmic punctae/vesicles in the papillary layer (PL), RMA and SMA (J, K) as well as in odontoblasts, including the site of emergence of their processes (L). Because not all molar sections include SMA, the images in C and O were taken from other sections of the molars that have been processed for IHC under the same conditions. Images in B, D, F–H, J–L, N and P are high magnification views of the indicated areas in A, E, I and M, respectively. Additional abbreviations: DP, dental pulp mesenchyme, PD, predentin/dentin; TA, transition-stage ameloblasts. Scale bars: 200 µm (A, E, I, M), 20 µm (B–D, F–H, J–L, N–P).

The distribution of CA proteins in tooth forming cells at this stage are summerized in Table S1. In both molar and incisor teeth, cells of the odontoblastic lineage and the rest of the dental pulp mesenchyme continued to express high levels of CA II/*Car2* and moderate levels of CAVI/*Car6* (Fig. 3A, D, M, P & Fig. S3I–L’). Remarkably, CA III staining was robust in odontoblasts in the roots of molars, whereas their counterparts in the crown showed nearly background staining (Fig. 3E & H). This peculiar pattern was clearly shown with immunofluorescence (Fig. S2AA’). At this stage of odontogenesis, odontoblasts expressed CA IV (Fig. 3I & L), CARP XI (Fig. 4E & H) and CA XIV (Fig. 4M & P) though with varying immunostaining intensities depending on the target. In contrast to the weak staining of newly differentiated odontoblasts at the bell stage (Fig. 2D’), odontoblasts displayed a net increase in CA IX immunoreactivity (Fig. 4A & D). Both CA XIII antibodies revealed strong punctate as well as some cytoplasmic staining in odontoblasts (Fig. 4L, Fig. S4H), and the punctate staining was also detectable in developing odontoblast processes which are cytoplasmic extensions of these cells (Fig. S4T).

In the epithelial part of both molars and incisors, as compared to secretory ameloblasts (Fig. S3A, D, K–L’), transition-stage ameloblasts and maturation-stage ameloblasts (MA) as well as the papillary layer (PL) displayed a net boost in CAII/*Car2* (Fig. 3A–C, Fig. S3II’, MM’ & data not shown) and CA VI/*Car6* (Fig. 3M–O, Fig. S3JJ’, NN’ & data not shown) expression levels. Whereas CA III immunostaining was weak in secretory ameloblasts (Fig. S3B), it became intense in MA, and the PL associated with ruffle-ended MA (RMA) was stained as well (Fig. 3E–G). Interestingly, in molars that had started root formation, a clear boost of CA III immunoreactivity took place in the occlusal part of the outer dental epithelium (Fig. 3E & Fig. S2AA’). Notably, amid differences in staining intensities between secretory ameloblasts (Fig. S3C, E & F) and MA, CA IV, CA IX and CARP XI immunostaining revealed dynamic subcellular distribution patterns of these proteins in MA. In this regard, the ruffle-ended MA (RMA) exhibited CA IV (Fig. 3J) and CARP XI (Fig. 4F) staining which mainly decorated their ruffled border, whereas the proteins were homogeneously distributed over the smooth-ended MA (SMA) (Fig. 3K & Fig. 4G). CA IX staining was either weak or strong in the ruffled border of the RMA (Fig. 4B & data not shown) but showed a homogeneous distribution over the SMA (Fig. 4C). Both CA XIII antibodies visualized strongly stained punctae and a moderate cytoplasmic staining in secretory ameloblasts and the stratum intermedium (Fig. S3G & Fig. S4T) as well as in MA and the PL (Fig. 4J, K & Fig. S4B, C). The homogeneous cytoplasmic CA XIII staining in secretory ameloblasts and odontoblasts in PFA-fixed specimens may be non-specific, since it was detected in negative controls (Fig. S2M). In addition, the robustness of the reaction product in intracytoplasmic vesicles may generate artefactual cytoplasmic staining as shown after using a high primary antibody concentration (Fig. S4F & G). It is noteworthy that this background staining was not observed in sections processed without tyramide amplification (used with all the other antibodies tested). Finally, CA XIV immunostaining was strong in both secretory ameloblasts (Fig. S3H) and MA (Fig. 4M–O).

**Figure 5:**
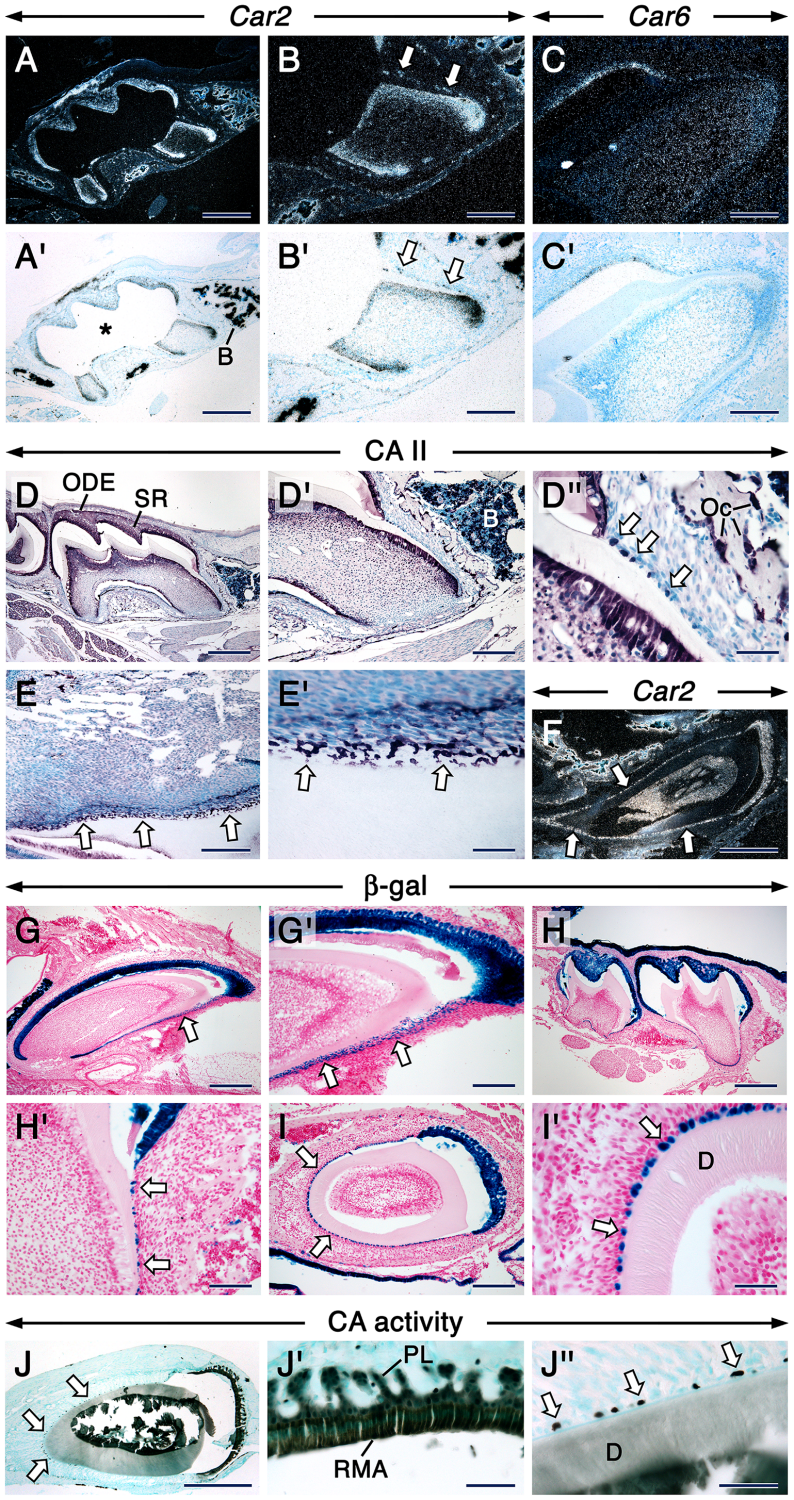
In situ hybridization, immunohistochemistry and histochemistry in postnatal teeth. In situ hybridization (ISH) showing expression of *Car2* (A, A’, B, B’) and *Car6* (C, C’) in sections at the level of roots from first molars and *Car2* in a sagittal section of a maxillary incisor (arrow in F). Signals in dark-field views appear as shiny dots (A–C, F) and robust signals appear as black dots in bright-field images (A’–C’). The epithelial rests of Malassez (ERM; arrows) are *Car2*-positive (B, B’) and in the incisor they display a lace-like network (F). Asterisk in A’ indicates an artifact subsequent to loss of dental pulp mesenchyme during processing. Immunoshistochemistry showing CA II-positive (dark magenta color) ERM (arrows) along the first molar’s root surface (D–D”). The ERM (arrows) form a CA-II-positive (E, E’) lace-like network at the surface of the incisor’s root analog as shown in a section tangential to the surface of the tooth. The ERM (arrows) are visualized by β-galactosidase histochemistry (dark blue color) in sagittal (G–H’) and transversal (I, I’) sections of molars (H, H’) and incisors (G, G’, I, I’) from *K14-Cre; R26R* reporter mice. The β–positive ERM form a lace-like network (G, G’). Histochemistry visualizing CA activity (blackish color) in ruffle-ended maturation-stage ameloblasts (RMA), the papillary layer (PL) as well as in the ERM (arrows) in an incisor’s section (J–J”). Images in BB’ and D’ are high magnification views of areas in AA’ and D, respectively. Images in D”, E’, G’, H’, I’, J’ and J” are high magnification views of areas in D’, E, G, H, I and J, respectively. Additional abbreviations: B, bone/bone marrow; ODE, outer dental epithelium; OC, osteoclasts; SR, stellate reticulum. Scale bars: 500 µm (AA’, D, F, G, H), 200 µm (B, B’, C;C’, D’, E, G’, I, J), 50 µm (D”, I’, J’, J”).

Remarkably, both incisors and molars displayed strong *Car2* signals (Fig. 5BB’, Fig. S3MM’) and CA II immunostaining (Fig. 5D–D”) in the epithelial rests of Malassez (ERM). These are remnants of Hertwig’s epithelial root sheath made of inner and outer dental epithelia located at the the surface of developing roots and root analog of molars and incisors, respectiveley [46], [52]–[55]. Previous studies in mammalian molar and premolar teeth [52], [53], [56]–[59] showed that the ERM form lacelike strands of interconnected epithelial cells. Here we show with tangential sections of mouse incisors that in this type of tooth too, at least at 12 dpp, the CA II/*Car2*-positive ERM form a network (Fig. 5EE’& F). The ERM associated with molar and incisor roots/root analog, including their lacelike network formation around the root analog of incisors, were well visualized after β-galactosidase histochemistry in sections from *K14-Cre; R26R* reporter mice which visualizes epithelial cells (Fig. 5G–I’). By contrast, weak CA VI/*Car6* signals and moderate punctate CA XIII staining were observed in cells of the periodontal ligament, including cells along the root surface, whereas only periodontal ligament fibroblasts showed CA XIV staining (Fig. 5CC’ & data not shown).

To determine whether the ERM show CA activity, we performed enzyme histochemistry. This clearly showed high levels of CA activity in these cells and, as expected, in MA as well as in the PL (Fig. 5J–J”).

### Cellular Distribution Patterns Suggest that CA XIII Protein Localizes in Lysosomes

**Figure 6:**
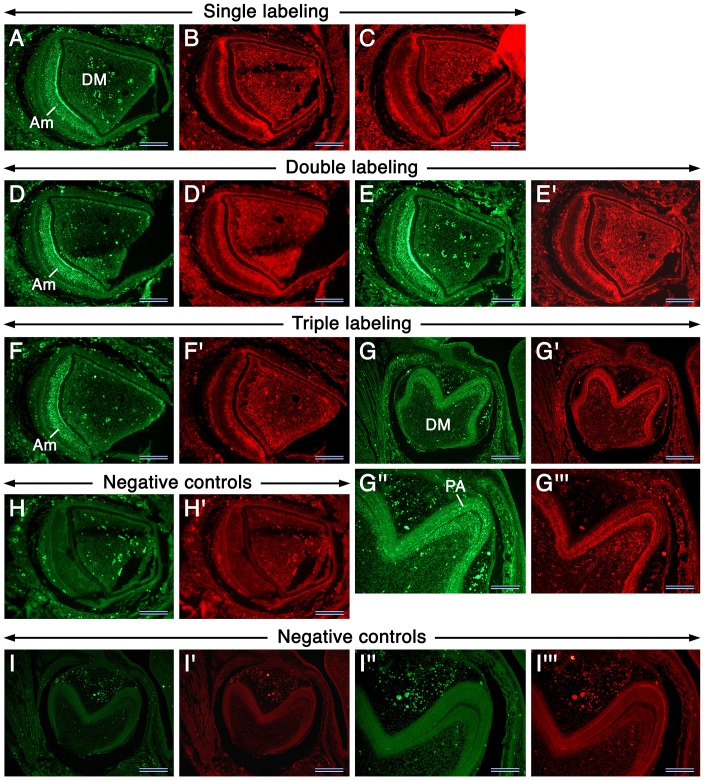
Immunofluorescence showing the distribution of CA XIII, LAMP-1 and LAMP-2 in developing teeth. Images of sections of incisors (A–F’; H, H’) and molars (G, G’, I, I’) at 1 dpp. Incisor sections after single immunostaining showing CA XIII (green fluorescence (GF) in A), LAMP-1 (red fluorescence (RF) in B) and LAMP-2 (RF in C). Incisor sections after double immunostaining showing CA XIII (GF in D) and LAMP-1 (RF in D’) or CA XIII (GF in E) and LAMP-2 (RF in E’). Incisor and molar sections after triple immunostaining showing CA XIII (GF in F and G) and LAMP-1+ LAMP-2 (RF in F’ and G’). Negative controls processed without the primary antibodies (H, H’, I, I’). The GF and RF spots are artifacts due to autofluorescence of red blood cells, somes areas in preameloblasts (PA) as well as areas with tissue folds. G”, G’’’, I” and I”’ are high magnifications views of areas in G, G’, I and I’, respectively. Abbreviations: Am, differentiating ameloblasts; DM, dental papilla mesenchyme. Scale bars: 200 µm (G–G’, I, I’), 100 µm (A–F’, H, H’, G”, G”’, I”, I”’).

One of the salient observations in this study was the localization of CA XIII in numerous intracellular punctae in developing teeth (this was also found in non-dental tissues and cells, data not shown). Because the CA XIII-positive punctae displayed different apparent sizes, we anticipated that these may represent CA XIII protein within lysosomes. To further elucidate this hypothesis, we made immunohistochemistry for LAMP-1 and LAMP-2, well-known integral membrane proteins of lysosomes [60]. We found that at different developmental stages, LAMP-1, LAMP-2 and CA XIII immunostaining displayed similar patterns of distribution in developing teeth (Fig. 6, Fig. S2, Fig. S4 & data not shown), which at postnatal stages visualized vesicles with different sizes, some of which were quite large, especially in maturation-stage ameloblasts (Fig. S4).

### CA Expression in Embryonic and Early Postnatal Non-dental Tissues

Since we processed embryo and head sections to analyse the distribution patterns of CAs during tooth development, we were able to uncover previously unknown tissues and cells displaying robust CA immunostaining during embryogenesis and shortly after birth (data not shown). At E12.5, in addition to CA III [15], the notochord and its derivative, the developing nucleus pulposus, showed robust CA II/*Car2* expression, the latter also displayed strong CA VI immunostaining. In addition, high levels of CAII/*Car2* were detected in brain and meningeal blood vessel endothelia, the developing choroid plexus (CP) and the developing eye, whereas CA VI was robustly expressed in the developing vomeronasal organ. Remarkably, robust CA IV expression was distinctly restricted to the epithelium of the anterior part of the developing tongue as well as to endothelia in the developing brain and meninges. However, after E14.5, CA IV protein in the tongue became restricted to a subset of taste bud cells. Finally, at E12.5 onwards, intense CA XIV immunoreactivity was visible in the developing CP, retina and telencephalon (dorsal pallium and medial pallium). With the exception of the notochord, which normally disappears after E12.5 leaving only its derivative the nucleus pulposus, those tissues continued to express the different CAs at later embryonic stages.

Other novel cephalic sites of CA expression were found shortly after birth. Remarkably, at 1 dpp anti-CA IV strongly labelled dendritically-shaped cells within the stroma of the ciliary body, the mesenchyme subjacent to the retinal pigmented epithelium (RPE), the Harderian gland’s mesenchyme, the stria vascularis of the inner ear as well as in the skin. In the latter, these cells were found in the dermis, epidermis as well as hair and whisker follicles. Given their sites of distribution in newborns [61], [62] and the fact that they were no longer detectable in the epidermis and dermis at later postnatal stages, we posit that these CA IV-positive cells are melanoblasts, precursors of melanocytes. At 1 dpp and 12 dpp, robust CA IX staining was found in the olfactory epithelium, vomeronasal organ and glands within the nasal cavity. At 1 dpp and 12 dpp, in addition to the retina, the developing iris, ciliary process, anterior lens epithelium as well as the RPE exhibited robust CA XIV immunostaining. CA XIV was also readily detectable in the corneal stroma and the mesenchyme subjacent to the RPE. Finally, at 12 dpp, we uncovered *Car2* expression in sebaceous glands and were able to to visualize strong CA II staining in a subset of olfactory neuron axons within the lamina propria as well as in the olfactory nerve.

## Discussion

### Dynamic Expression Patterns of Carbonic Anhydrases during Tooth Formation

Carbonic anhydrases (CAs) in teeth, with a special focus on maturation-stage ameloblasts (MA), have earlier been studied in tissue sections by histochemistry and immunohistochemistry as well as in dental tissue extracts by PCR and Northern blot analyses [26], [30]–[35]. However, when and in which dental cells are CAs expressed during odontogenesis is hitherto unknown. Our systematic study revealed interesting dynamic expression patterns of eight CA enzymes in murine developing teeth.

The ameloblastic lineage is unique as it undergoes extraordinary changes during the different stages of odontogenesis. The actively proliferating preameloblasts as well as their progenitors, the cells of the inner dental epithelium, are first separated from the dental papilla mesenchyme by a basement membrane. After disappearance of the basement membrane following predentin secretion by newly differentiated odontoblasts, preameloblasts withdraw from the cell cycle and differentiate into secretory ameloblasts (SA). The latter undergo a tremendous growth in size and develop apical cytoplasmic extensions known as Tomes’ processes [63]. After completion of enamel secretion, SA differentiate into maturation-stage ameloblasts after a short transition stage during which they decrease their height and lose their Tomes’ processes. Maturation-stage ameloblasts (MA) play a crucial role during amelogenesis since they degrade and reabsorb a major part of enamel proteins, a pre-requisite for maturation of enamel into a highly organized mineralized tissue. These cells undergo a unique series of morphological changes by modulating between two phenotypes: (1) the ruffle-ended ameloblasts (RMA), which have their plasma membrane deeply infolded at their apical pole; and (2) the smooth-ended ameloblasts (SMA), which have a smooth apical plasma membrane [49], [50], [64], [65]. These two cell types are also distinguished functionally, with the RMA being more actively involved in endocytosis than their SMA counterparts [66], [67].

Importantly, the enamel extracellular matrix undergoes pH shifts. With essentially neutral values (pH 7.2) during the secretory stage, during the maturation stage the pH oscillates between acidic, near neutral and even alkaline values, with low pH values at the level of the RMA and alkaline conditions at the level of SMA [67]. These changes entail a stringent regulation of extracellular pH in which the Na^+^/H^+^ exchanger (NHE1), the vacuolar-type H^+^-ATPases (V-H-ATPases), the cystic fibrosis transmembrane conductance regulator (CFTR) as well as proteins involved in bicarbonate transport, including CAs, the electrogenic Na^+^-bicarbonate co-cotransporter (NBCe1) and the anion exchanger 2 (AE2), have been proposed to play critical roles [26], [32], [67], [68]. AE2 [69], [70] and CFTR [71] deficient mice display defects in enamel maturation, and AE2 [68]–[70], NHE1 [68] and V-H-ATPases [32], [68], [72] proteins have been shown to be expressed by MA. NBCe1 protein is expressed in the papillary layer [68], and human mutations of *SLC4A4* [73], [74] as well as mice with deletions in *Slc4a4* [75], the gene encoding NBCe1, have enamel anomalies. However, AE2, NBCE1 and NHE1 were also detected at the secretory stage of amelogenesis, with the two former present in the developing papillary layer (PL) and the latter in secretory ameloblasts [68].

Discrepancies exist as to the expression of CA II protein in the PL in the rat incisor; this has been found to be both CA II-positive [33] and CA II-negative [32], [68]. In this study, we clearly demonstrated that the PL in both molars and incisors displays high levels of expression of CA II/*Car2* and shows robust CA activity just like MA. Furthermore, we showed that in addition to CA II/*Car2* and CA VI/*Car6*, MA express CA III, CA IV, CA IX, CARP XI, CA XIII and CA XIV with some differences in their subcellular localizations, not only between the different isoenzymes but also between the RMA and SMA.

A previous study using RT-PCR showed that several CAs are expressed in extracts of the epithelial enamel organ at the secretory stage of amelogenesis, and that *Car2*, *Car6*, *Car9*, *Car11* and *Car13* displayed the highest levels of expression [35], however the identity of the dental epithelial cells that express these isoforms was hitherto unknown. In addition, discrepancies existed as to the expression of CA II in secretory ameloblasts, which were found to be devoid of CA II protein [33] as well as of CA activity in general [31], whereas extracts of the epithelial enamel organ at the secretory stage of amelogenesis showed expression of *Car2* mRNA [34], [35]. Here, we show that CA II/*Car2* as well as CA III, CA VI/*Car6*, CA IX, CARP-XI, CA XIII and CA XIV are expressed at varying levels in secretory ameloblasts. In addition, CA IX and CA XIII were expressed in the stratum intermedium, and substantial CA XIII protein amounts were detected in the inner dental epithelium and preameloblasts. Finally, CA II/*Car2*, CAVI/*Car6*, CARP XI and CA XIV were also detectable in preameloblasts, while robust CA III in the occlusal portion of the outer dental epithelium became evident in molars with developing roots, i.e. molars that have initiated the eruption process.

The inner dental epithelium, preameloblasts as well as the stratum intermedium at the bell stage of odontogenesis are highly proliferating cells [25], [44], whereas secretory ameloblasts and MA are highly active in secretion and reabsorption of enamel components, respectively. Taken together, our findings suggest that during tooth development CAs are likely involved in different aspect of the biology of the ameloblastic lineage and cells of the stratum intermedium, including proliferation, survival, differentiation, secretory as well as resorptive activities.

Carbonic anhydrase activity has been detected histochemically in preodontoblasts and odontoblasts [30], [31]. However, which isoenzymes are expressed in the odontoblastic lineage is hitherto unknown. We showed that not only differentiated odontoblasts but also undifferentiated preodontoblasts as well as the rest of dental papilla cells express a set of CAs. Of the most remarkable findings are the high expression levels of CA II/*Car2*, CA XIII-stained punctae as well as relatively lower levels of CA VI/*Car6* expression in the dental papilla mesenchyme during the early bell stage of odontogenesis. In fact, CA XIII was expressed in both the dental epithelium and mesenchyme, starting at the earliest stages of tooth development. At later developmental stages (12 dpp), CA II/*Car2* and CA VI/*Car6* continued to be expressed at high and relatively lower levels, respectively, in preodontoblasts, odontoblasts and dental pulp cells in both tooth crown/crown analog and roots/root analog. A peculiar finding was the asymmetrical distribution of CA III in both molar and incisor teeth. Newly differentiated and more mature odontoblasts (no staining in preodontoblasts) in the roots of molars as well as the root analog part of incisors displayed a robust staining as compared to their counterparts in the crown and crown analogs, which were weakly stained. The biological significance of this pattern of CA III staining is at present unclear, however it could be secondary to the topographical differences of root and crown odontoblasts and influences from nearby cells during their development. Odontoblasts also displayed high levels of CA XIII and CA XIV as well as moderate levels of CARP-XI during all developmental stages, starting at the bell stage. By contrast, after being weak in newly differentiated odontoblasts, CA IX immunoreactivity became stronger in odontoblasts. With the exception of CA II/*Car2*, CA VI/*Car6* and CA XIII, the other CAs were not detected in preodontoblasts and dental papilla mesenchyme. These data suggest that different CAs may be involved in proliferation, differentiation, biosynthetic as well as secretory activities in odontoblasts and their precursors.

Taken together, our findings show dynamic expression patterns of CAs in tooth-forming cells from the earliest stages until near-completion of tooth development, suggesting a tight control of their expression. Thus, CAs may perform crucial and specific functions during odontogenesis. Remarkably, children affected by carbonic anhydrase II deficiency syndrome have been reported to display tooth defects, ranging from delayed dentition, peg-shaped teeth as well as enamel hypoplasia [76], [77]. However, to our knowledge, no tooth defects have been described in *Car2*, *Car4*, *Car6* and *Car14* mutant mice. Whether these do have subtle tooth defects that have been overlooked or are exempt of dental anomalies due to functional compensation by other isoenzymes or other reasons is at present unknown.

From our findings and numerous previous studies [1], [3]–[5], it is clear that CAs are ubiquitous enzymes found in nearly all mammalian tissues and cells with high metabolic activities. Amongst the numerous physiological processes in which they are involved, CAs contribute at determining the levels of protons and bicarbonate and thereby regulate intracellular and extracellular volume, keep in check intracellular and extracellular pH shifts and help in disposing of excess CO_2_ (as bicarbonate) produced by high cellular metabolic activities in cells that also express bicarbonate transporters [1], [3], [5]. CO_2_ is membrane permeant. Cells thus express CAs, which by catalysing the reversible conversion of carbon dioxide and water into bicarbonate and protons prevent the slow interchange between CO_2_ and HCO_3_^−^ that could impede H^+^ shuttling in the cytosol and across the cell membrane [78]. CAs with an extracellular catalytic site, including CA IV, CA IX, CA XII and CA XIV, catalyse the reaction at the cell surface extracellularly, but CA IV seems also to be active intracellularly [79]. Other CAs such as CA I, CA II, CA III and CA XIII function intracellularly [78].

What could be the function of CA IV, CA IX and CA XIV in ameloblasts and odontoblasts? Because these cells are metabolically active as they perform secretory (newly differentiated odontoblasts, odontoblasts and secretory ameloblasts) and resorptive (MA) functions, membrane-bound and cytosolic CAs may perform functions extracellularly and intracellularly, similar to those found in other tissues with high metabolic activities. In this regard, CA IV and CA XIV in brain as well as CA XIV in retina are crucial for extracellular pH regulation [28], [80], [81]. In the stomach, it is likely that CA IX is required not only for secretory activities but also for regulating proliferation [23], whereas in brain absence of CA IX leads to neuronal vacuolar degeneration [24]. CA IX and CA XII are misexpressed in a range of tumors, are induced by hypoxia, a condition found in solid tumors, and are associated with cancer progression. These enzymes and possibly also CA II may provide a unique environment for tumor cells, enabling them to outcompete their normal counterparts by contributing to the acidification of the extracellular milieu and increasing intracellular pH. One way to do so is by forming a so-called metabolon with bicarbonate transporters [2].

### Carbonic Anhydrases in the Epithelial Rests of Malassez

An intriguing finding in this study was the high levels of expression of CA II/*Car2* in the epithelial rests of Malassez (ERM), remnants of the bilayered Hertwig’s epithelial root sheath (HERS), known to be present at the surface of developing molar roots and incisor’s root analog [46], [52]–[55]. In addition, like in molar teeth [52], [53], [56]–[59], we uncovered the existence of a network of such remnants at the surface of the mouse incisor’s root analog, which were also CAII/*Car2*-positive. Furthermore, we showed that ERM exhibit robust CA activity. Some of HERS cells have been shown to be incorporated into the developing cementum, a mineralized extracellular matrix that covers the roots/root analog [58], [82], and to be involved in formation of acellular and cellular cementum [59]. The exact function of the ERM is to date unknown. Clinical and developmental biology studies suggested that ERM are crucial for homeostasis and maintenance of the width of the periodontal ligaments and protect the tooth against ankylosis as well as root resorption [58]. More recently, it has been suggested that the network of ERM may constitute a signaling center for molar root development [59]. It is also believed that the ERM generate cysts and tumors in the jaws when abnormally activated [57]. The occurrence of CAs and of CA activity in ERM naturally raises the question as to their function, which needs further studies.

### Subcellular Localization of Carbonic Anhydrases

In contrast to CA II, CA III and CAVI immunostaining, which strongly decorated the entire cytoplasm of maturation-stage ameloblasts (MA), CA IV, CA IX and CARP XI staining was strong in the ruffled border of RMA, whereas it was more diffusely distributed in SMA. These differences suggest the occurrence of a dynamic distribution of these proteins during the modulation cycle of MA.

At all developmental stages studied, a remarkable constant was the presence of CA XIII-laden intracytoplasmic punctae, not only in dental cells but also in cells of other cephalic structures as well as in the adult kidney. Importantly, MA as well as the papillary layer (PL) showed strong CA XIII staining in large intracytoplasmic vesicles. These vesicles may represent lysosomes, since MA and the PL are known to be rich in large lysosomes, owing to their resorptive activities [83]–[85]. Immunostaining for LAMP-1, LAMP-2 and CA XIII in developing teeth disclosed similar patterns of intracellular vesicular distribution in tooth-forming cells. Furthermore, immunofluorescence showed that, to a large extent, LAMP-1, LAMP-2 and CA XIII staining of intracytoplasmic vesicles overlaps, suggesting that CA XIII may be a lysosomal enzyme. Our LAMP-1 and LAMP-2 staining was similar to previous findings of LAMP-1 expression in the ameloblastic lineage of mouse teeth [86].

At present, although we favor CA XIII localization in lysosomes, we do not rule out its presence in other components of the endolysosomal system as well, since early and late endosomes express low and high levels of LAMP proteins, respectively. It is unlikely that the CA XIII vesicular staining portrays the protein being degraded in lysosomes or portions of cytoplasm containing CA XIII engulfed in autolysosomes for the following reasons: First, our previous immunoshistochemical studies of numerous proteins never disclosed a vesicular staining for non-lysosomal proteins. Second, as discussed above, the cytoplasmic CA XIII staining is likely an artefact. Of note, a vesicular/punctate intracytoplasmic staining in cultured cells (www.proteinatlas.org) was observed with another CA XIII antibody [87]. Importantly, there is precedent for the occurrence of CA activity and protein in lysosomes, though the identity of the lysosomal CA was hitherto undisclosed [88]–[91]. Thus, in light of those studies and our findings, we suggest that CA activity in lysosomes is likely contributed by the activity of CA XIII. Lysosomal membrane proteins and lysosomal hydrolases can be sorted to lysosomes through two routes, one is direct starting at the trans Golgi network level towards the endosomal system, the other is indirect through the plasma membrane whereupon proteins are endocytosed [60], [92]. The direct and best characterized lysosomal sorting of lysosomal hydrolases involves the mannose-6 phosphate receptor pathway [60], [92]. How CA XIII is targeted to lysosomes remains to be deciphered.

Lysosomes are vital organelles, and disrupted lysosomal biogenesis and function generate several human diseases [60], [92], [93]. Remarkably, unlike the other cytosolic carbonic anhydrases, mouse (m) CA XIII was found to be resistant to inhibition by the physiological anion bicarbonate, and similar to human (h) CA II and hCA V, mCA XIII showed an impressive resistance to inhibition by chloride [94]. It is noteworthy that the luminal concentration of Cl^−^ is known to increase along the endocytic pathway to reach an estimated value greater that 80 mM within lysosomes [95]. Progressive luminal acidifications are known to take place along the endolysosomal pathway to reach pH 4.5–5 values in lysosomes. The mechanisms regulating intralysosomal pH are however, still unclear [93], [95], [96]. Because optimal acidic pH is crucial for lysosomal function, we propose that mCA XIII, being able to function in the presence of high chloride concentrations, acts as a buffering system for optimal intralysosomal pH. Since carbonic anhydrases would provide an ‘inexpensive’ way as a buffering system, and despite early findings on the occurrence of CA activity and protein in lysosomes [88]–[91], it is intriguing that these enzymes are not included in studies describing ion transport and pH regulation in lysosomes. Our findings are thus of importance not only from the cell biology point of view, but are also expected to direct interest towards CAs in diseases linked to lysosomal dysfunction.

## Supporting Information

Figure S1 **Positive controls.** Immunohistochemistry in sections from E12.5 (B, C) and E14.5 (A) embryos as well as from adult mice (D–J). CA II is expressed by the chroid plexus (CP) and blood vessels in the developing brain and meninges (A). The notochord (N) and developing nucleus pulposus (NP) express CA III (B). CA III expression in the rostral-most extension (NR; in the head at the level of the pituitary) of the notochord (C). Sections of the kidney showing the distribution of CA IV (D), CA XIII (I) and CA XIV (J). Section of the submandibular salivary gland showing the distribution of CA VI (E). Sections of the stomach showing the distribution of CA IX after immunostaining with the rabbit anti-CAIX (F) and goat anti-CA IX (CAIX*; G). Section of cerebellum showing expression of CARP XI (H) in Purkinje neurons (P). Intracellular punctae/vesicles in kidney tubule cells show strong CA XIII staining (I). Scale bars: 500 µm (C), 200 µm (A), 100 µm (D–H, J), 20 µm (H).
(TIF)

Figure S2 **Distribution of CA III, LAMP-2 and negative controls.** Sections of a molar (A) and incisor (B) at 12 dpp after anti-CA III immunofluorescence. A’ is a high magnification view of A. Strong CA III immunostaining in maturation-stage ameloblasts (MA), the papillary layer (PL), the outer dental epithelium (ODE) at the occlusal part of the tooth, odontoblasts (in the root of the molar (arrow in A’) and in the root analog (RA) of the incisor. Odontoblasts (Od) in the molar’s crown and incisor’s crown analog (CA) are virtually CA III-negative. Section of 1 dpp incisor at the level of secretory ameloblasts after double staining for CA XIII and LAMP-2 (C, D). Negative control (for sections after immunohistochemistry with the LAMP-1 and LAMP-2 antibodies at 12 dpp) without the primary antibody (E). Sagittal sections of molars at 12 dpp used as negative controls for primary antibodies made in goat [without tyramide amplification (F, G)] and rabbit (H–J). Asterisks indicate non-specific staining of the enamel (G) and dentin (H) matrices. Sections of a molar (K, L) and incisor (M) at 1 dpp used as negative controls (without the primary antibody) for goat anti-CA XIII staining with tyramide amplification. Negative control (for staining tooth sections with the goat CA XIII antibody in post-natal teeth) section processed without the primary antibody (N–P). Additional abbreviations: RMA, ruffle-ended maturation-stage ameloblasts, SA, secretory ameloblasts, PA, preameloblasts. Scale bars: 500 µm (A), 200 µm (A’, B, E, F, H, K, N), 100 µm (C, D), 50 µm (I, J), 20 µm (G, L, M, O, P).
(TIF)

Figure S3 **Immunohistochemistry and in situ hybridization in sections from postnatal molars and incisors.** Sections of third molars at the level of the principal cusps (A–H) showing the distrubution of CA proteins (indicated on the panels) in secretory ameloblasts (SA) as visualized (dark magenta color) by immunohistochemistry. SA exhibit strong CA XIII (G) and CA XIV (H) immunostaining, with the former strongly decorating intracytoplasmic punctae/vesicles. Moderate staining portrays CA II (A), CA VI (D) and CA IX (E) in SA. The latter is also detected in the stratum intermedium (SI). The asterisks indicate non-specific reactions in the enamel matrix. In situ hybridization showing the expression patterns of *Car2* and *Car6* as indicated on the panels. The sites of expression are portrayed by shiny dots in dark-field images (I–N) and high levels of expression appear as black areas in bright-field images (I’–N’). Sections of second molars (I–J’). Transversal sections of maxillary incisors at the level of secretory (K–L’) and maturation-stage (M–N’) ameloblasts. Maturation-stage (MA) and transition-stage (TA) ameloblasts show robust expression of *Car2* and *Car6* as compared to SA. The papillary layer (PL) is rich in *Car2* transcripts. Preodontoblasts (pOd), newly differentiated odontoblasts (nOd) and odontoblasts (Od) show strong and moderate expression levels of *Car2* and *Car6*, respectively. Additional abbreviation: B, bone/bone marrow; DP, developing dental pulp; ERM, epithelial rests of Malassez. Scale bars: 500 µm (I–J’), 200 µm (K–N’), 50 µm (A–H).
(TIF)

Figure S4 **Immunohistochemistry showing the distribution of CA XIII, LAMP-1 and LAMP-2 in postnatal teeth.** Sections of molars showing the distribution of CA XIII (A–H, Q, T), LAMP-1 (I–P, R, U) and LAMP-2 (S, V–X). CA XIII detection after immunohistochemistry (IHC; dark magenta color indicates the positive sites) using a high (1∶1500; shown in A–D, Q and T) or a low dilution (1∶500, shown in E–H) of the primary antibody made in goat. LAMP-1 distribution following IHC with a high (1∶50000, shown in I–L, R and U) and low (1∶40000 shown in M–P) dilutions of the primary antibody. The CA XIII, LAMP-1 and LAMP-2 display similar patterns of intracytoplasmic vesicular staining which becomes clear with high dilution of the primary antibodies. Both the tall (B, J, N, X) and short (C, F, K) ruffle-ended maturation-stage ameloblasts (RMA) as well as the smooth-ended maturation-stage ameloblasts (SMA), odontoblasts (Od) and the papillary layer (PL) display numerous CA XIII-, LAMP-1- and LAMP-2-positive vesicles of different sizes. Sections of the third molar (Q–S) showing similar distribution patterns of CA XIII, LAMP-1 and LAMP-2 in intracytoplasmic punctae/vesicles in secretory ameloblasts (SA), the stratum intermedium (SI) and odontoblasts, including odontoblast processes (Arrowheads in T, U and V). Images in B, D, F, H, J–L, N–P, T–V, X) are high magnification views of areas in A, E, I, M, Q, R, S and W. The images in C and G are from other sections of the same molar processed under the same conditions. Scale bars: 200 µm (A, E, I, M,W), 100 µm (Q–S), 20 µm (B–D, F–H, J–L, N–P, T–V, X).
(TIF)

Table S1 **Carbonic anhydrase protein distribution in the developing tooth.**
(XLSX)
