# Supplementary figures and images for: Expression Patterns and Subcellular Localization of Carbonic Anhydrases Are Developmentally Regulated during Tooth Formation
Source: PLoS One. 2014 May 1;9(5):e96007. doi: 10.1371/journal.pone.0096007 (PMC4006843; doi:10.1371/journal.pone.0096007)

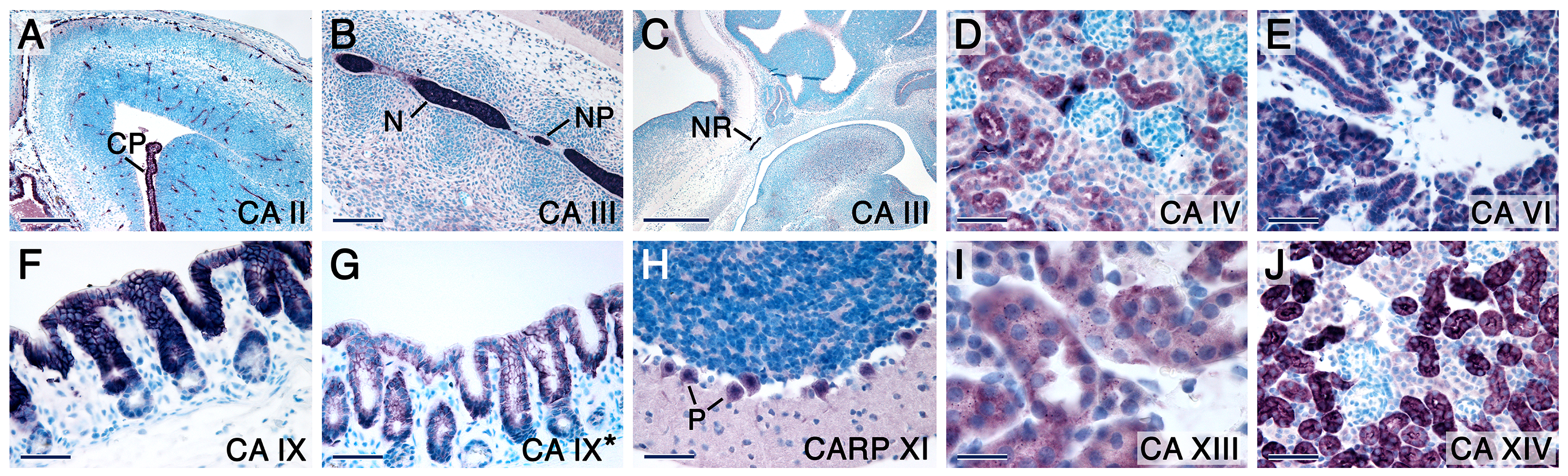

Supplement: Figure S1 — Positive controls. Immunohistochemistry in sections from E12.5 (B, C) and E14.5 (A) embryos as well as from adult mice (D–J). CA II is expressed by the chroid plexus (CP) and blood vessels in the developing brain and meninges (A). The notochord (N) and developing nucleus pulposus (NP) express CA III (B). CA III expression in the rostral-most extension (NR; in the head at the level of the pituitary) of the notochord (C). Sections of the kidney showing the distribution of CA IV (D), CA XIII (I) and CA XIV (J). Section of the submandibular salivary gland showing the distribution of CA VI (E). Sections of the stomach showing the distribution of CA IX after immunostaining with the rabbit anti-CAIX (F) and goat anti-CA IX (CAIX*; G). Section of cerebellum showing expression of CARP XI (H) in Purkinje neurons (P). Intracellular punctae/vesicles in kidney tubule cells show strong CA XIII staining (I). Scale bars: 500 µm (C), 200 µm (A), 100 µm (D–H, J), 20 µm (H). (TIF) [file pone.0096007.s001.tif]

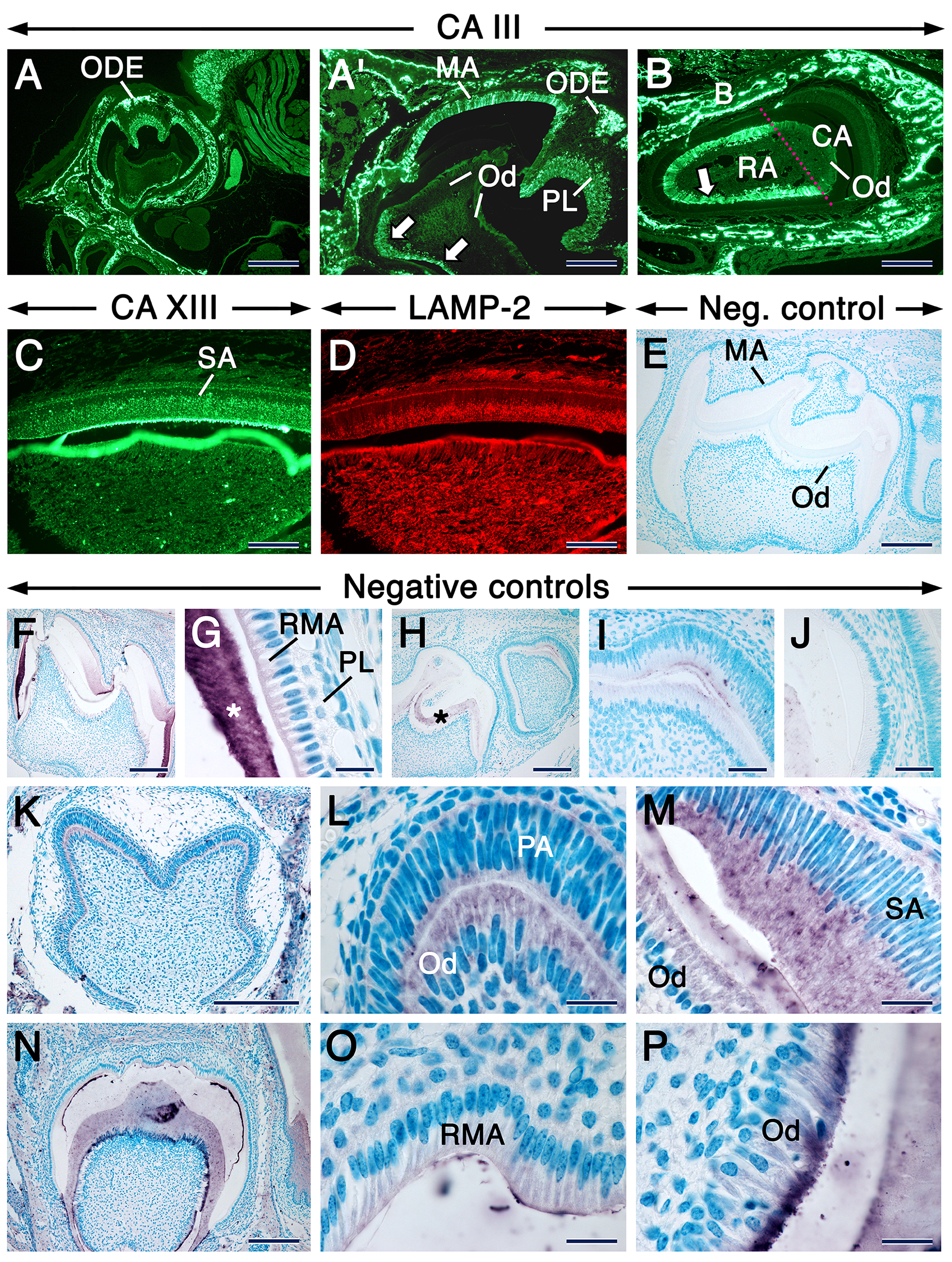

Supplement: Figure S2 — Distribution of CA III, LAMP-2 and negative controls. Sections of a molar (A) and incisor (B) at 12 dpp after anti-CA III immunofluorescence. A’ is a high magnification view of A. Strong CA III immunostaining in maturation-stage ameloblasts (MA), the papillary layer (PL), the outer dental epithelium (ODE) at the occlusal part of the tooth, odontoblasts (in the root of the molar (arrow in A’) and in the root analog (RA) of the incisor. Odontoblasts (Od) in the molar’s crown and incisor’s crown analog (CA) are virtually CA III-negative. Section of 1 dpp incisor at the level of secretory ameloblasts after double staining for CA XIII and LAMP-2 (C, D). Negative control (for sections after immunohistochemistry with the LAMP-1 and LAMP-2 antibodies at 12 dpp) without the primary antibody (E). Sagittal sections of molars at 12 dpp used as negative controls for primary antibodies made in goat [without tyramide amplification (F, G)] and rabbit (H–J). Asterisks indicate non-specific staining of the enamel (G) and dentin (H) matrices. Sections of a molar (K, L) and incisor (M) at 1 dpp used as negative controls (without the primary antibody) for goat anti-CA XIII staining with tyramide amplification. Negative control (for staining tooth sections with the goat CA XIII antibody in post-natal teeth) section processed without the primary antibody (N–P). Additional abbreviations: RMA, ruffle-ended maturation-stage ameloblasts, SA, secretory ameloblasts, PA, preameloblasts. Scale bars: 500 µm (A), 200 µm (A’, B, E, F, H, K, N), 100 µm (C, D), 50 µm (I, J), 20 µm (G, L, M, O, P). (TIF) [file pone.0096007.s002.tif]

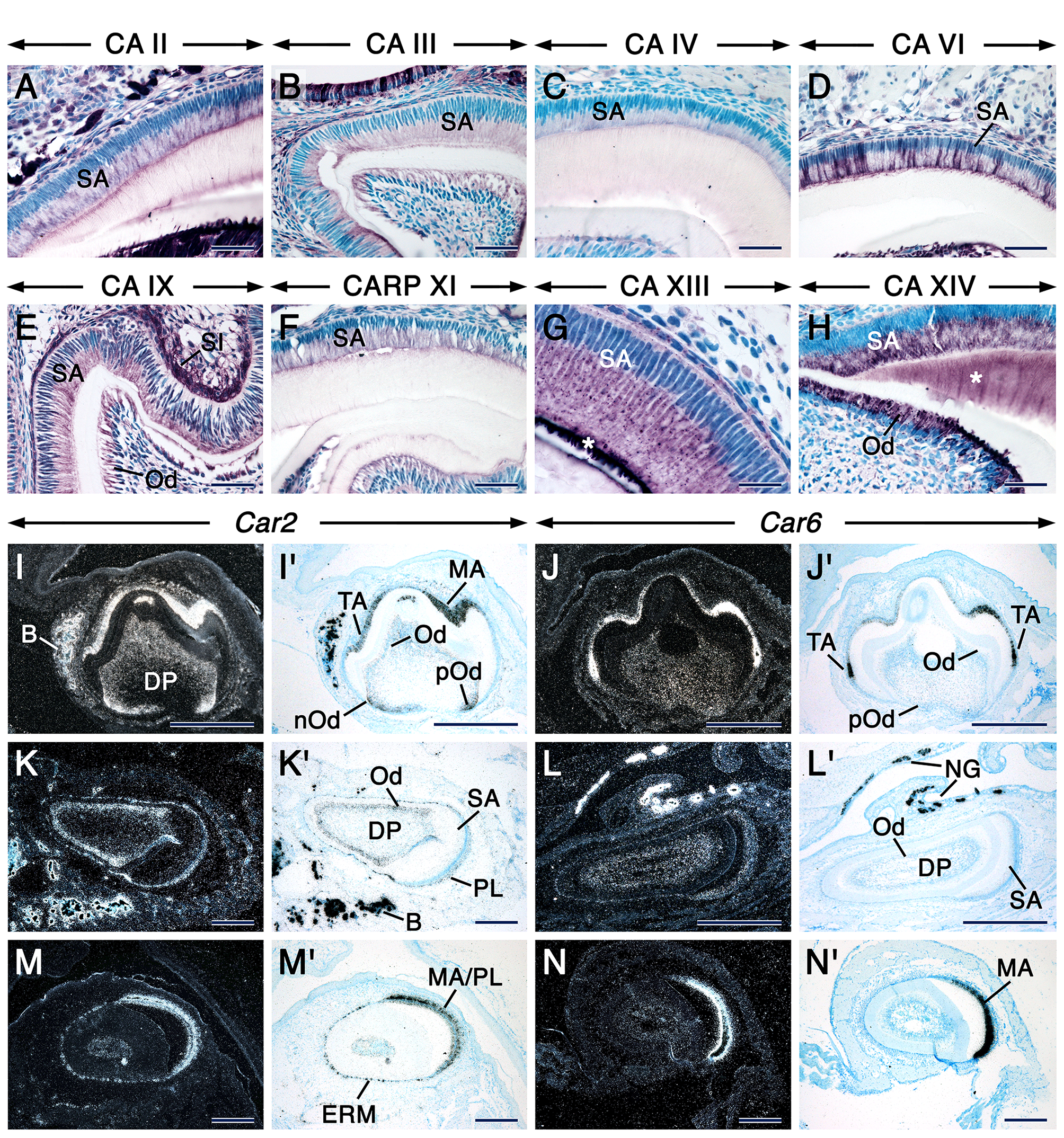

Supplement: Figure S3 — Immunohistochemistry and in situ hybridization in sections from postnatal molars and incisors. Sections of third molars at the level of the principal cusps (A–H) showing the distrubution of CA proteins (indicated on the panels) in secretory ameloblasts (SA) as visualized (dark magenta color) by immunohistochemistry. SA exhibit strong CA XIII (G) and CA XIV (H) immunostaining, with the former strongly decorating intracytoplasmic punctae/vesicles. Moderate staining portrays CA II (A), CA VI (D) and CA IX (E) in SA. The latter is also detected in the stratum intermedium (SI). The asterisks indicate non-specific reactions in the enamel matrix. In situ hybridization showing the expression patterns of Car2 and Car6 as indicated on the panels. The sites of expression are portrayed by shiny dots in dark-field images (I–N) and high levels of expression appear as black areas in bright-field images (I’–N’). Sections of second molars (I–J’). Transversal sections of maxillary incisors at the level of secretory (K–L’) and maturation-stage (M–N’) ameloblasts. Maturation-stage (MA) and transition-stage (TA) ameloblasts show robust expression of Car2 and Car6 as compared to SA. The papillary layer (PL) is rich in Car2 transcripts. Preodontoblasts (pOd), newly differentiated odontoblasts (nOd) and odontoblasts (Od) show strong and moderate expression levels of Car2 and Car6, respectively. Additional abbreviation: B, bone/bone marrow; DP, developing dental pulp; ERM, epithelial rests of Malassez. Scale bars: 500 µm (I–J’), 200 µm (K–N’), 50 µm (A–H). (TIF) [file pone.0096007.s003.tif]

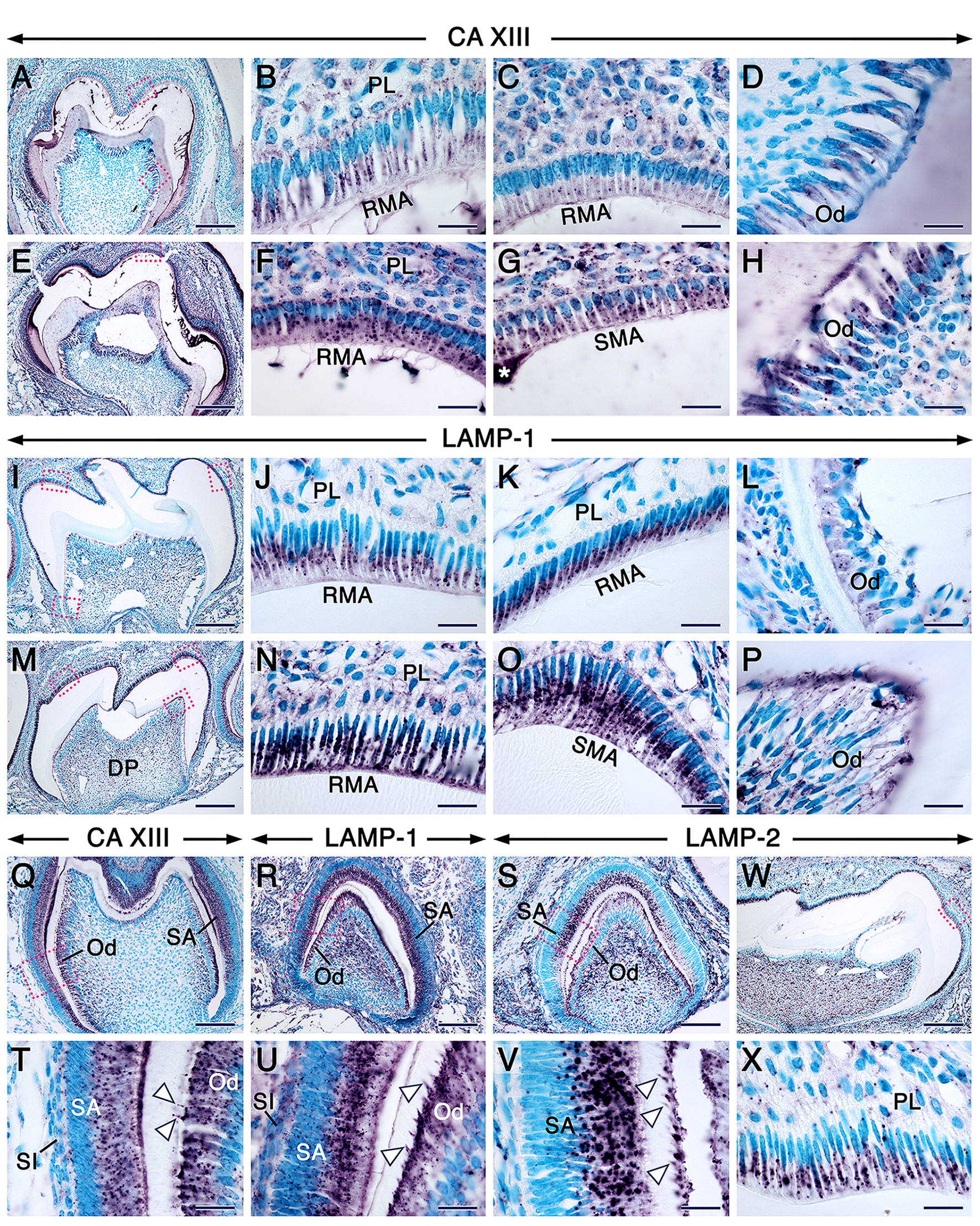

Supplement: Figure S4 — Immunohistochemistry showing the distribution of CA XIII, LAMP-1 and LAMP-2 in postnatal teeth. Sections of molars showing the distribution of CA XIII (A–H, Q, T), LAMP-1 (I–P, R, U) and LAMP-2 (S, V–X). CA XIII detection after immunohistochemistry (IHC; dark magenta color indicates the positive sites) using a high (1∶1500; shown in A–D, Q and T) or a low dilution (1∶500, shown in E–H) of the primary antibody made in goat. LAMP-1 distribution following IHC with a high (1∶50000, shown in I–L, R and U) and low (1∶40000 shown in M–P) dilutions of the primary antibody. The CA XIII, LAMP-1 and LAMP-2 display similar patterns of intracytoplasmic vesicular staining which becomes clear with high dilution of the primary antibodies. Both the tall (B, J, N, X) and short (C, F, K) ruffle-ended maturation-stage ameloblasts (RMA) as well as the smooth-ended maturation-stage ameloblasts (SMA), odontoblasts (Od) and the papillary layer (PL) display numerous CA XIII-, LAMP-1- and LAMP-2-positive vesicles of different sizes. Sections of the third molar (Q–S) showing similar distribution patterns of CA XIII, LAMP-1 and LAMP-2 in intracytoplasmic punctae/vesicles in secretory ameloblasts (SA), the stratum intermedium (SI) and odontoblasts, including odontoblast processes (Arrowheads in T, U and V). Images in B, D, F, H, J–L, N–P, T–V, X) are high magnification views of areas in A, E, I, M, Q, R, S and W. The images in C and G are from other sections of the same molar processed under the same conditions. Scale bars: 200 µm (A, E, I, M,W), 100 µm (Q–S), 20 µm (B–D, F–H, J–L, N–P, T–V, X). (TIF) [file pone.0096007.s004.tif]
